# Predation risk triggers copepod small-scale behavior in the Baltic Sea

**DOI:** 10.1093/plankt/fbaa044

**Published:** 2020-10-13

**Authors:** Klas Ove MÖller, Michael St. John, Axel Temming, Rabea Diekmann, Janna Peters, Jens Floeter, Anne F Sell, Jens-Peter Herrmann, Dominik Gloe, Jörn O Schmidt, Hans H Hinrichsen, Christian MÖllmann

**Affiliations:** Institute of Coastal Research, Helmholtz-Zentrum Geesthacht, Max-Planck-Strasse 1, 21502 Geesthacht, Germany; Institute of Marine Ecosystem and Fishery Science, University of Hamburg, Große Elbstrasse 133, 22767 Hamburg, Germany; National Institute of Aquatic Resources, Technical University of Denmark, Kemitorvet, 2800 Kongens Lyngby, Copenhagen, Denmark; Institute of Marine Ecosystem and Fishery Science, University of Hamburg, Olbersweg 24, 22767 Hamburg, Germany; University of Applied Sciences Bremerhaven, An der Karlstadt 8, 27568 Bremerhaven, Germany; Deutsches Zentrum für Marine Biodiversitätsforschung, Senckenberg am Meer, Südstrand 44, 26382 Wilhelmshaven, Germany; Institute of Marine Ecosystem and Fishery Science, University of Hamburg, Große Elbstrasse 133, 22767 Hamburg, Germany; Institute of Marine Ecosystem and Fishery Science, University of Hamburg, Große Elbstrasse 133, 22767 Hamburg, Germany; Thünen Institute, Institute of Sea Fisheries, Herwigstraße 31, 27572 Bremerhaven, Germany; Institute of Marine Ecosystem and Fishery Science, University of Hamburg, Olbersweg 24, 22767 Hamburg, Germany; Institute of Marine Ecosystem and Fishery Science, University of Hamburg, Große Elbstrasse 133, 22767 Hamburg, Germany; International Council for the Exploration of the Sea, Science Committee, H. C. Andersens Boulevard 44-46, 1553 Copenhagen V, Denmark; Kiel University, Center for Ocean and Society, Neufeldtstrasse 10, 24118 Kiel, Germany; GEOMAR, Helmholtz Centre for Ocean Research, Marine Ecology, Marine Evolutionary Ecology, Düsternbrooker Weg 20, 24105 Kiel, Germany; Institute of Marine Ecosystem and Fishery Science, University of Hamburg, Große Elbstrasse 133, 22767 Hamburg, Germany; Center for Earth System Research and Sustainability (CEN), University of Hamburg, Große Elbstraße 133, 22767 Hamburg, Germany

**Keywords:** diel vertical migration, individual behavior, predator avoidance, video plankton recorder, zooplankton

## Abstract

Predators not only have direct impact on biomass but also indirect, non-consumptive effects on the behavior their prey organisms. A characteristic response of zooplankton in aquatic ecosystems is predator avoidance by diel vertical migration (DVM), a behavior which is well studied on the population level. A wide range of behavioral diversity and plasticity has been observed both between- as well as within-species and, hence, investigating predator–prey interactions at the individual level seems therefore essential for a better understanding of zooplankton dynamics. Here we applied an underwater imaging instrument, the video plankton recorder (VPR), which allows the non-invasive investigation of individual, diel adaptive behavior of zooplankton in response to predators in the natural oceanic environment, providing a finely resolved and continuous documentation of the organisms’ vertical distribution. Combing observations of copepod individuals observed with the VPR and hydroacoustic estimates of predatory fish biomass, we here show (i) a small-scale DVM of ovigerous *Pseudocalanus acuspes* females in response to its main predators, (ii) *in-situ* observations of a direct short-term reaction of the prey to the arrival of the predator and (iii) *in-situ* evidence of pronounced individual variation in this adaptive behavior with potentially strong effects on individual performance and ecosystem functioning.

## INTRODUCTION

Predators not only have direct effects on their prey biomass, but can also induce important indirect effects such as trophic cascades or influences on diversity, production and nutrient cycling (Greig and McIntosh, 2006; Trussell *et al*., 2006; Schmitz *et al*., 2008, 2010; Strong and Frank, 2010). Among these indirect effects of predation are non-consumptive effects on behavior of prey organisms (Preisser *et al*., 2009). Generally, herbivores and other intermediate trophic level species need to balance the trade-off between maximizing energy or nutrient intake and minimizing predation risk (Werner and Peacor, 2003; Preisser *et al*., 2005). Such an adaptive foraging behavior hence can have important implications for ecosystem functioning, e.g. by affecting transfer efficiencies between trophic levels. Furthermore, predator avoidance behavior may force prey species into unfavorable environments in terms of food availability or physical habitat variables such as temperature, salinity and oxygen (Sainmont *et al*., 2012). Hence, from an evolutionary perspective species in danger of predation need to respond adaptively to balance fitness gains from foraging with fitness losses from predation (Mangel and Clark, 1988; Lima and Dill, 1990; Lima, 1998).

An important adaptive behavior in aquatic ecosystems is diel vertical migration (DVM) that has been demonstrated for diverse organisms from protists up to fish (Bollens and Frost, 1989; Kaartvedt *et al*., 2007; Cohen and Forward, 2009; Ringelberg, 2010). DVM has major ecological consequences at the individual, population, community and ecosystem level as it has been shown in freshwater and marine ecosystems (Dodson *et al*., 1997; Ramirez-Llodra *et al*., 2010; Bollens *et al*., 2012; Sainmont *et al*., 2012). Furthermore, it plays an important role in global biogeochemical cycles by modifying and transporting inorganic and organic material throughout the water column (Robinson *et al*., 2010). DVM of planktonic organisms in lakes and oceans represents one of the most widespread and massive migration of animals on Earth (Williamson, 2011).

A number of causes are discussed to induce DVM including light conditions, food availability and temperature (Dupont *et al*., 2009; Ringelberg, 2010) or trade-offs between those (Loose and Dawidowicz, 1994). For zooplankton such as copepods, the most wide-spread animal group in the world ocean, there is general consensus that predator avoidance is a major driver for DVM (Lampert, 1989; Bollens and Frost, 1989, 1991; Hays, 2003; Bollens *et al*., 2011). In principle, DVM behavior may be caused and regulated by ultimate and proximate aspects, respectively (Lampert *et al*., 2003, Ringelberg and Van Gool, 2003). The most common pattern of zooplankton DVM involves an avoidance of shallower depth during daylight and, hence, visual predators such as planktivorous fish (e.g. Zaret and Suffern, 1976; Frost, 1988; Buskey *et al*., 2011). The role of predation in DVM and, hence adaptive foraging behavior of an ecologically important group such as marine copepods is unequivocal (Sainmont *et al*., 2012), but direct observations of the relative behavior of predator and prey in natural environments are still rare. Traditional net sampling methods often fail to resolve the spatial and temporal scales necessary for observing DVM in plankton and parallel predator biomass measures are frequently lacking.

Observing variability in individual plankton behavior in vast natural environments such as the ocean is especially difficult since net sampling typically provides only population mean differences over larger spatial scales. Hence, aggregated group-level data may blur and misrepresent individual behavior, a phenomenon called the “ecological fallacy” (Wakefield and Shaddick, 2006; Clark *et al*., 2011). Experimental studies in behavioral ecology have demonstrated strong variation in behavior within single populations which may be due to individual personality and plasticity in response to environmental cues (Biro and Dingemanse, 2009; Dingemanse *et al*., 2009). Stage-specific variation in predator avoidance has been shown for zooplankton populations in relation to feeding history, size, reproductive status and pigmentation (Baumgartner *et al*., 2011; Holliland *et al*., 2012; Lönnstedt *et al*., 2012). A wide range of diversity and plasticity in morphological, physiological and behavioral phenotypes, has been observed both between-species as well as within-species (Cohen and Forward, 2009; Kaartvedt *et al*., 2011). Freshwater *Daphnia* have been observed to exhibit individual swimming behavior during DVM triggered by predator cues (Dodson *et al*., 1997) and adults often swim further than juveniles (Dodson, 1990). Especially trophic interactions in natural environments seem to occur at the individual rather than at the population level (Kiørboe, 2008). Therefore, investigating predator–prey interactions at the individual level seems essential for a better understanding of zooplankton dynamics. This is important since predator hunting strategy and, consequently, prey escape strategy can be viewed as key functional traits that partly control the top-down interactions in ecosystems (Legreneur *et al*., 2012).

We applied an optical underwater sampling gear, the video plankton recorder (VPR), which allows in contrast to traditional net sampling the investigations of individual, diel adaptive behavior of zooplankton in response to predators in the natural oceanic environment. We studied the zooplanktonic *Pseudocalanus acuspes,* a key species for the ecosystem functioning of the pelagic foodweb of the Central Baltic Sea (Möllmann *et al*., 2009). The population of *P. acuspes* has recently suffered from climate induced changes in the physical environment (Möllmann *et al*., 2003; Möller *et al*., 2015). Furthermore, overfishing the local top predator cod (*Gadus morhua*) caused a strong predation pressure on *P. acuspes* by planktivorous small pelagic fish (Casini *et al*., 2008; Möllmann *et al*., 2008). The clupeids sprat (*Sprattus sprattus*) and herring (*Clupea harengus*) are almost exclusively dominating the group of planktivorous fish in the simple trophic structure of the Baltic Sea (Rudstam *et al*., 1994) and, hence, are the main predator of the copepod community including *P. acuspes*. Up to 80% of the annual zooplankton production are consumed by adult sprat and herring (Arrhenius and Hansson, 1993) and Casini *et al*. (2008) suggested a top-down control of sprat on zooplankton in the open Baltic Sea.

Combining observations of copepod individuals with the VPR and hydroacoustic estimates of predatory fish biomass, we here show (i) a small-scale DVM of ovigerous *P. acuspes* females in response to its main predator sprat and herring, (ii) *in-situ* observations of a direct short-term reaction of the prey to the arrival of the predator and (iii) *in-situ* evidence of strong individual variation in this adaptive behavior with potentially strong effects on individual performance and ecosystem functioning.

## METHOD

### The model species

**Fig. 1:**
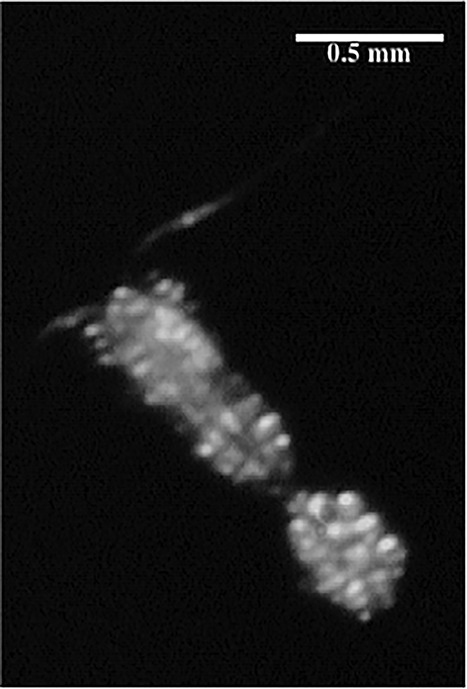
Example image of a *P. acuspes* female with egg sac.

We studied DVM of the calanoid copepod *P. acuspes* that is an important component in the Baltic ecosystem (Möllmann *et al*., 2009; Fig. 1) as are other species of the genus *Pseudocalanus* spp. in most areas of the world ocean (McLaren and Corkett, 1978; Hopcroft and Kosobokova, 2010). While in older studies for the Baltic Sea *P. acuspes* was also referred to as *Pseudocalanus minutus* (Dahmen, 1995), *Pseudocalanus elongatus* (Möllmann *et al*., 2000) or *P. minutus elongatus* (Hernroth, 1985) a genetic approach by Holmborn *et al*. (2011) proved that *P. acuspes* is the only *Pseudocalanus* species living in the Baltic Sea. Therefore, we assumed that all egg sac carrying individuals identified in the VPR images were members of this species. A key feature of its life-cycle is an ontogenetic vertical migration, i.e. its resident depth increases with individual age represented by developmental stage (Renz and Hirche, 2006). Copepodite stages and adult *P. acuspes* reside in deep waters specifically in the layer of highest salinity, the so-called halocline (Renz and Hirche, 2006; Hansen *et al*., 2006). This habitat provides them with concentrated food through marine snow aggregates (Möller *et al*., 2012) and a salinity level that allows successful reproduction (Renz and Hirche, 2006). However, the downside of this habitat is a high predation risk since populations of small pelagic and planktivorous fish (i.e. sprat and herring) feed here during daytime (Köster and Schnack, 1994). Hence, our model species within its special habitat is ideal for studying adaptive behavior because *P. acuspes* individuals need to deal with the trade-off between maximizing energy or nutrient intake and minimizing predation risk.

### Data acquisition with the video plankton recorder

**Fig. 2:**
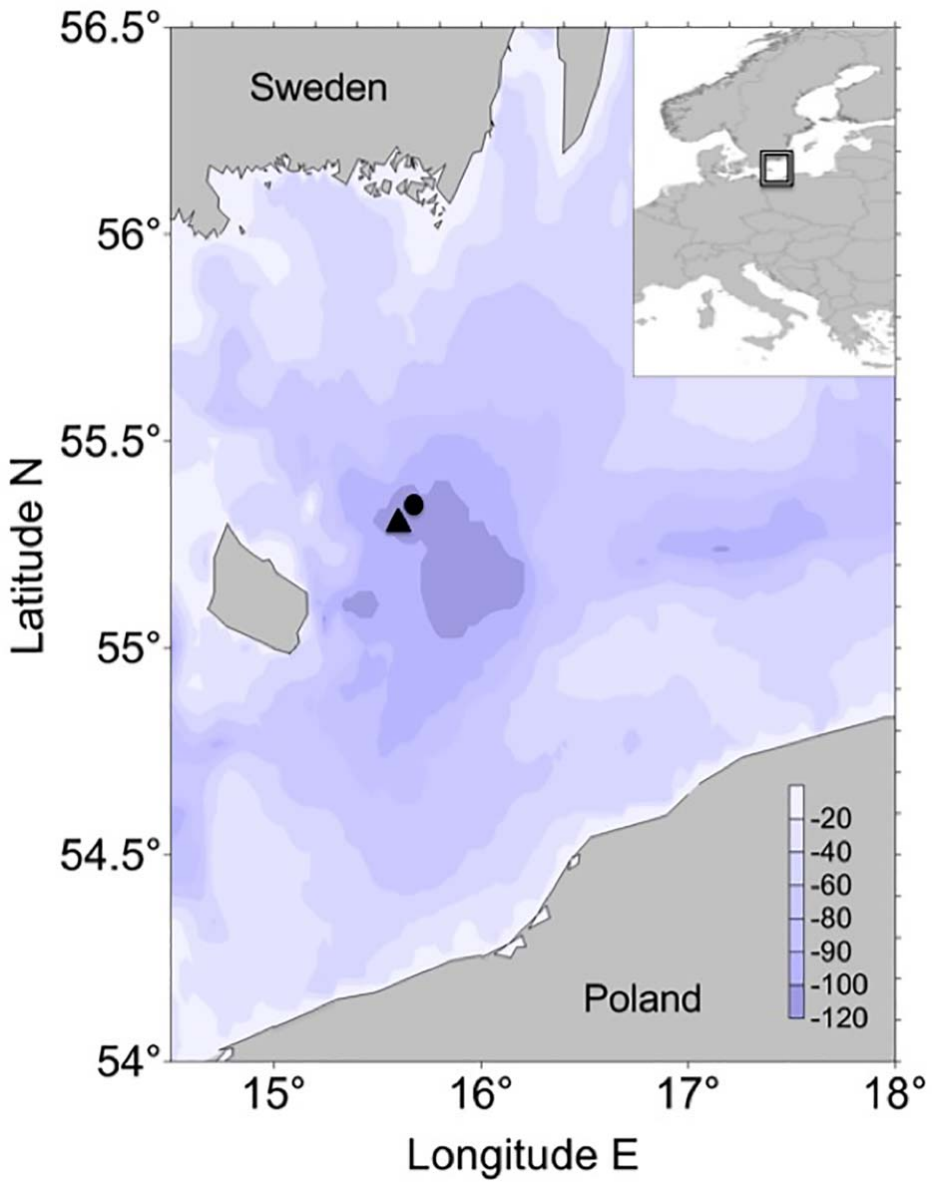
Map of the Central Baltic Sea with the study area in the Bornholm Basin; the black dot indicates the area of the VPR sampling location in 2002 and the black triangle the VPR and multi-net sampling location in 2009. The upper right panel gives an overview of the geographic location of the sampling area. The color coded legend reflects water depth.

We obtained high-resolution images of plankton using the VPR during field campaigns in April 2002 and May 2009 aboard the research vessel “Alkor” in the Bornholm Basin of the Central Baltic Sea (Fig. 2). The VPR (Seascan) is an underwater camera system towed by a research vessel. Our VPR was equipped with a high-resolution digital camera (Pulnix TM-1040), which records 25 image frames s^−1^. We used a camera setting with a field of view of 0.7 x 0.7 cm and a calibrated image volume of 1.45 mL in 2002 and 1.10 mL in 2009. The camera was set to the largest magnification (f-zoom) due to the small particle and plankton size in the Baltic Sea. Illumination for the camera is provided by a strobe (Seascan/20 W Hamamatsu Xenon bulb) with a pulse duration of 1 μs, which was synchronized with the camera shutter. Additionally, the VPR was equipped with hydrographic and environmental sensors for temperature and salinity (CTD) (Falmouth Scientific Inc.) as well as fluorescence (Seapoint Inc., model SCF). Additionally, oxygen data were obtained using a CTD-probe equipped with an oxygen sensor measuring vertical profiles after each VPR haul.

**Fig. 3:**
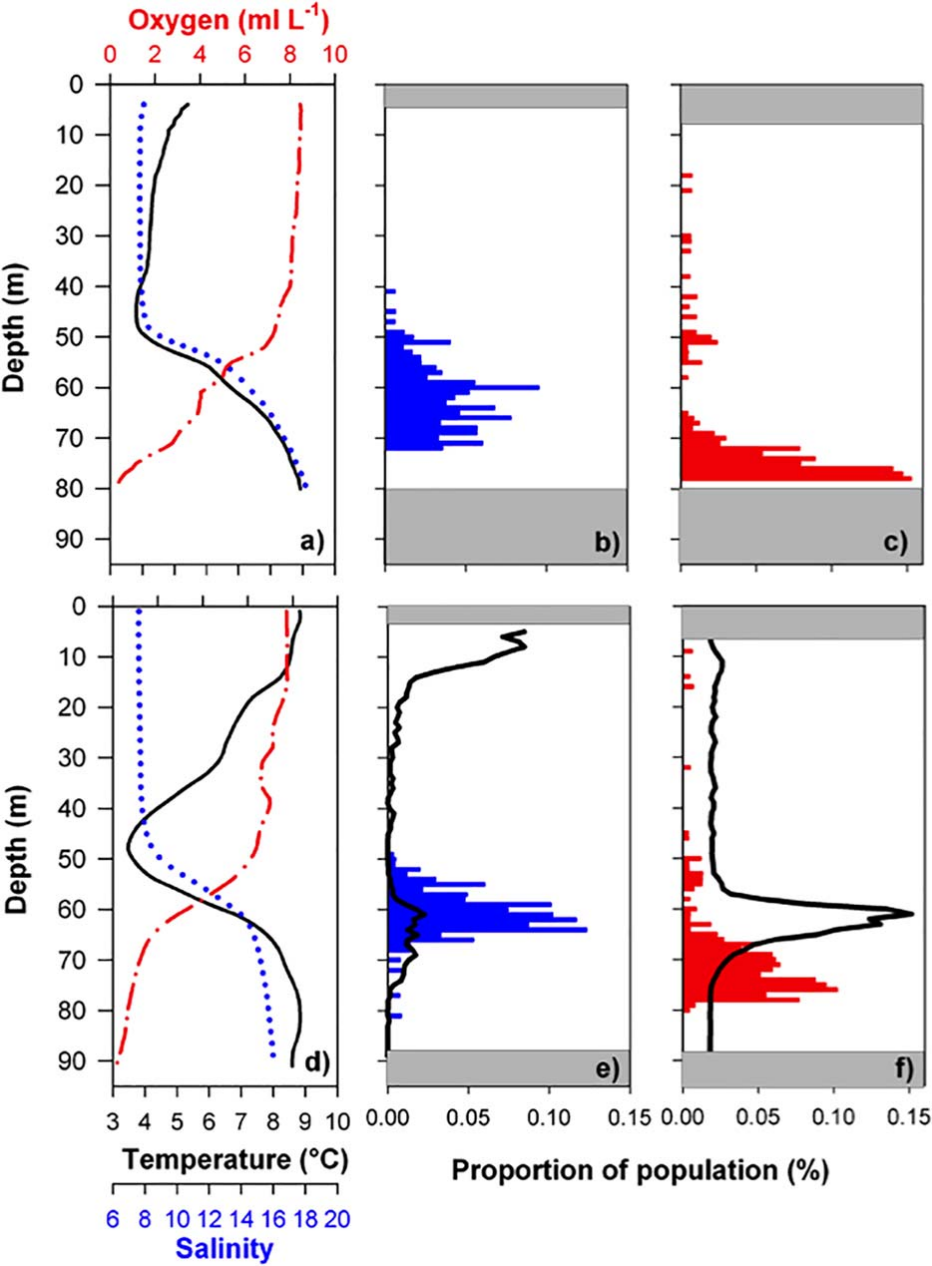
Environmental data and vertical day and night distribution patterns of ovigerous *P. acuspes* in 2002 (upper row) and 2009 (lower row): (a) and (d) Vertical profiles of temperature (°C, black line), salinity (blue line) and oxygen (mg ml^−1^, red dot-dashed line) averaged for each VPR tow track in 1 m depth intervals; (b, c) and (e, f) Vertical average nighttime (b and e, blue bars) and daytime (c and f, red bars) proportional distribution (%) of ovigerous *P. acuspes* in 1 m depth bins sampled with the VPR. Black line indicates the vertical distribution of clupeid fish (proportional NASC [m2nmi-2]); gray areas visualize the limits of the average sampling depth representing the ranges to surface and bottom without sampling.

The VPR was mounted on an equipment rack with a v-fin depressor and undulated continuously from near bottom to near surface, hence, surveying the whole water column. In order to exclude the influence of turbulence in the ship’s wake and to maintain a safe distance from the bottom, sampling was only conducted between ~5 m below the surface and 8 m above the bottom, but varied between the VPR surveys (see Fig. 3). The VPR was towed between 11 p.m. and 11 a.m. covering the full night/day transition from April 25th and 26th in 2002. In 2009, four separate VPR hauls were conducted between May 17th (4 p.m.) and 18th (7 p.m.) covering day-, night-time and the day/night transition. The gear was towed with a speed of 1–1.5 m s^−1^, covering a distance of 115 km and a total sampling volume of 15 65l in 2002 and a distance of 91.2 km and a total sampling volume of 12 42l in 2009. Recorded images and sensor data were sent in real time to an onboard unit via a fiber optic cable. Plankton and other particle images were extracted from each image frame as regions of interest (ROIs) using the Autodeck image analysis software (Seascan) and saved to the computer hard drive as TIFF files. Each ROI was tagged using a timestamp to allow merging with the hydrographic parameters that were written to a separate logfile.

Assigning VPR images to different copepod species is not always possible and still true for most data from *in-situ* imaging systems currently applied (Lombard *et al*., 2019). However, the Baltic Sea is due to a strong stratification and low species diversity and the resulting small amount of trophic linkages (Sandberg *et al*., 2000) a very unique and in our case ideal study area. The copepod community of the Baltic Sea consists of only four main species: *Pseudocalanus acuspes*, *Temora longicornis*, *Acartia bifilosa* and *Acartia longiremis* (Hansen *et al*., 2006; Renz and Hirche, 2006; Schmidt, 2006), which show different preferences in regard to their vertical distribution. *T. longicornis* as well as *Acartia* spp. dwell within the upper 30 m of the water column (Hansen *et al*., 2006) near the thermocline, whereas *P. acuspes* resides in deeper layers within the range of the halocline (Hansen *et al*., 2006). This strong spatial separation has also been observed and ground-truthed by comparing VPR observations and plankton net samples in a previous study (Möller *et al*., 2012). Furthermore, since most ROIs did not allow discrimination of copepods to the species level, we here only used ovigerous *P. acuspes* females in our analysis since these are best identified due to their distinct shape caused by attached egg sacs (Fig. 1). This condition provided us with the unique opportunity to generate small-scale distribution data that were not only species, but also sex and maturity stage specific and leaves us very confident that all individuals used and presented in this study are *P. acuspes*. In the following, we refer to ovigerous females only as *P. acuspes* females.

### Hydroacoustic fish biomass recording

We measured the biomass and vertical distribution of the planktivorous, clupeid fish population (i.e. sprat and herring) using hydroacoustic techniques during the cruise in 2009. During spring, our study area is inhabited by high clupeid abundances (Hoziosky *et al*., 1989; Stepputtis, 2006). In contrast to cod and other fishes only clupeids have been described to perform DVM in the Baltic Sea. Sprat and herring are visual predators and spend the day in deeper water layers near the halocline and the night at the surface (Cardinale *et al*., 2003; Nilsson *et al*., 2003). They form schools during dawn, migrate towards deeper water layers to avoid predators (Österblom *et al*., 2006) and spend the daylight hours in deep waters feeding on zooplankton (Stepputtis, 2006). The research vessel was equipped with a Simrad echosounder EK60 using a hull-mounted 38-kHz split-beam transducer. Hydroacoustic measurements were performed according to standard procedures for Baltic Sea acoustic surveys (ICES, 2001). Calibration settings of the echosounder and data processing were conducted using the Sonardata ECHOVIEW 4.9 software (Sonardata ECHOVIEW, 2010) applying a volume backscattering coefficient threshold of *S_V_* –60 dB, which is the standard threshold for clupeids (i.e. sprat and herring) in the Baltic (ICES, 2015). The water column was integrated with a vertical resolution of 1 m to allow direct comparisons to the vertical distribution of *P. acuspes*. Hydroacoustic recording was conducted the day following the VPR sampling which we assume does not introduce a major bias since the vertical pelagic fish distribution and behavior appears to be quite stable during spring in the study area (Stepputtis *et al*., 2011).

### Numerical analyses

We were interested in diel differences in the depth distribution of *P. acuspes* females. Thus, all VPR data were separated into day- (5 a.m. to 9 p.m.) and night-time (10 p.m. to 4 a.m.) values. Sampling times at dusk or dawn when copepods presumably migrate vertically were excluded from the analysis. Abundances of *P. acuspes* females were averaged for each hour of sampling and 1 m depth intervals. The center of the copepod distribution was calculated as the median depth and the corresponding upper and lower quartiles as variability measures. A modified two-sample Kolmogorov–Smirnov test was applied to test the null hypothesis of equal day and night depth distributions (Solow *et al*., 2000).

A modified test statistic *W* was applied

$$ W=\frac{\max \left(\left|{P}_{jk}-{P}_{jk}\right|\right)}{{\left(\sum_{k=j}^k{\left({p}_{jk}-{p}_{jk}\right)}^2\right)}^{\frac{1}{2}}} $$

with *P_jk_* being the sample cumulative distribution function of depth for the sample *j* and *p_jk_* the relative abundance of sample *j* in depth bin *k*. The significance of *W* was assessed by a randomization scheme underlying Fisher’s exact test of independence in a contingency table. During randomization, both samples were pooled and divided at random into two samples of sizes *n_1_* and *n_2_*. Row sums and column sums need to be the same as from the original samples. Eventually, W was calculated for 10^5^ permutations of the dataset and the significance level was estimated by the proportion of simulated *W*-values larger than the observed values. All calculations were performed within the statistical environment of R, version 2.14.2 (R Development Core Team, 2012).

As an index of vertical location of planktivorous fish, hourly weighted mean depths (WMD) (Bollens and Frost, 1989) were computed as: WMD = $\big(\big(\sum{n}_i{d}_i\big)/\sum{n}_i\big)$, where *n_i_* is the fish density expressed as nautical area scattering coefficient (NASC) of the population in depth stratum *i* with midpoint depth *d_i_*. The same index was computed for all female *P. acuspes* individuals, grouped together from both sampling periods to obtain the mean diel distribution during the day. Since *P. acuspes* individuals showed variable vertical distribution patterns during daytime, the mean of the hourly nighttime WMD was used as a threshold to compute average lower and upper WMD for each daytime hour.

We further investigated within-population variability of DVM. We tested for deviations from unimodality in the migratory behavior of the female *P. acuspes* population in response to their predators. We used Hartigans´ dip test (Hartigan and Hartigan, 1985) implemented in the dip function in the R diptest library. The test is very conservative and the distribution of the test statistic is based on asymptotic and empirical samples relative to a uniform distribution (Bestelmeyer *et al*., 2011). The power of the test (for α = 0.05) is 80% when sample size = 50. Since our sample sizes were much larger we used *P*-values ≤ 0.01 to indicate statistically significant evidence for deviations from unimodality.

## RESULTS

### Physical environmental habitat

As background information for our investigations on DVM of *P. acuspes* females, we recorded their physical habitat simultaneously to the VPR sampling (Fig. 3a and d). In upper water layers, the seasonal thermocline was visible in 2009, but not in 2002, which was due to the earlier cruise date in 2002. In deeper water layers, we clearly observed the permanent halocline at ~50–60 m depths, representing the typical habitat of *P. acuspes* females. Oxygen and salinity depth profiles were almost identical in both years showing a decline in oxygen levels below the halocline to almost complete oxygen deficiency. Below the permanent halocline temperature increased to ~9°C at greater depth, which is characteristic for the Central Baltic Sea.

### Average DVM patterns of prey and predator

The average water column abundance of observed ovigerous *P. acuspes* females integrated over all 1 m sampling bins was similar in both years with 0.3 ind. L^−1^ during night and a lower abundance of 0.23 ind. L^−1^ during day. Overall 388 and 524 individual *P. acuspes* females were observed with the VPR in 2002 and 2009, respectively. We recorded maximum abundance (average depth interval concentrations) of 2.14 (day) and 1.52 ind. L^−1^ (night) in 2002, and slightly lower values in 2009 with 1.45 (day) 2.82 ind. L^−1^ (night).

We found evidence of a DVM behavior in *P. acuspes* females during both sampling campaigns (Fig. 3b, c, and e, f), with a significantly deeper mean distributions during daytime (K-S test modified for patchy distributions; *P* < 0.001 for both years). Weighted median depth of the copepods was 75 and 63 m in 2002, and 72 and 61 m in May 2009 for day and night, respectively. Variability of the mean vertical distribution is generally low with deviations from the median (weighted upper and lower quartiles) of 5 and 7 (day) and 9 and 5 m (night) for 2002 and 2009, respectively. During daytime, the spread of the distribution was increasing and hence, individuals were observed between the surface and 50 m depth. We found the deeper daytime distribution of *P. acuspes* females related to the presence of planktivorous fish in the shallower habitats around the halocline. Planktivorous fish themselves performed an extended DVM being close to the surface during night (average WMD night = 23.8 m) and in and below the halocline during daytime (average WMD day = 58.5 m) (Fig. 3e and f). These planktivores form schools during dawn, migrate towards deeper water layers to avoid predators (e.g. Karlsson *et al*., 1999; Österblom *et al*., 2006) and spend the daylight hours in deep waters where they feed on zooplankton (Köster and Schnack, 1994; Stepputtis, 2006). Sprat are assumed to start their migration back to the surface into warmer waters at dusk and the individuals spend the night in surface layers.

### Within population variability in *P. acuspes* female DVM

**Fig. 4:**
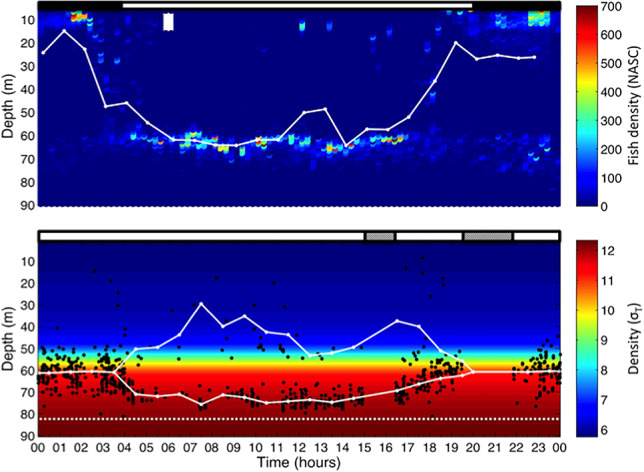
Vertical distribution of fish and ovigerous *P. acuspes*: (upper panel) Acoustic diel echogram showing the vertical distribution of clupeid fish. Density is given color-coded as NASC in [m2nmi-2]. White line indicates hourly WWD of vertical fish distribution. Bar on top represents day- (white) and night-time (black); (lower panel) Vertical distribution of individual ovigerous *P. acuspes* females (black dots) in relation to an averaged schematic density profile (*σ_T_*). Solid white lines indicate hourly WWD of all individuals (nighttime) and deviations from this (daytime). Bar on top of panel indicates VPR sampling (white) and data gaps (gray shaded). White dashed line indicates the maximum depth where individuals occurred.

In a refined analysis, we made use of the special ability of the VPR to identify individual *P. acuspes* and investigated whether the DVM was performed unidirectionally by all members of the population or whether within-population variability exists. In order to obtain an almost complete 24-hour cycle, we combined observations from both sampling campaigns into one dataset. Although vertical day- and night-time profiles were significantly different between 2002 and 2009 (K-S test, *P*_day_ < 0.05; *P*_night_ < 0.01), WMDs were comparable between years (see above). Following all 912 sampled female *P. acuspes* along the time-axis reveals a striking immediate response of the zooplankton population to the DVM of their predators (Fig. 4). During night, nearly all copepods dwelled uniformly in or around the halocline between 50 and 70 m. In response to the downward migration of the fish predators at sunrise, most individual zooplankter migrated into deeper water depths and aggregated in 70–80 m (max. average WMD = 71.7 m), where they remained during the day. Unexpectedly, a smaller part of the population avoided the predator population by moving in the opposite direction towards shallower depths (also visible in Fig. 3f).

Hence, our observations demonstrate substantial within population variability in DVM of *P. acuspes*. The results of the visual inspection is supported by a daytime vertical distribution that is significantly different from unimodality (Hartigans´ dip test: *P* < 0.01). Rather, we observed a bimodal vertical distribution with the “shallower fraction” of the copepod population displaying more variability than the “deeper fraction.” Eventually, at sunset, the predator population returns towards the surface and female *P. acuspes* return to concentrate unimodally in the center of their usual halocline habitat.

### Potential consequences of predator avoidance behavior

**Fig. 5:**
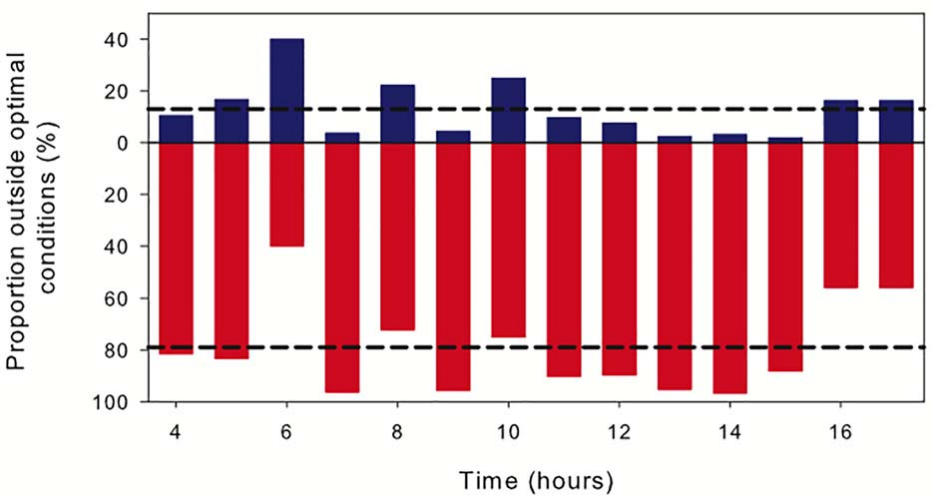
Proportion of individual *P. acuspes* outside the preferred habitat. Blue bars indicate proportion (%) of individuals above (adverse salinity), red bars below (adverse oxygen) the depth representing the preferred habitat; black dashed lines indicate the mean daytime proportion (%) of individuals above and below the preferred habitat, respectively.

The results of our study revealed an adaptive behavior in a copepod species with a fast response to the presence of its predator population that let them leave their preferred habitat during daytime. Hence, we assumed the nighttime distribution to represent optimal conditions for *P. acuspes* females in terms of hydrographic and food conditions while leaving these can lead to reduced growth, reproductive success and survival. As an indication of potential negative effects of the DVM, we calculated the proportion of the population that is migrating out of the optimal conditions as a consequence of predator presence (Fig. 5).

Based on our observations, we determined the distribution range between 55 and 65 m (WMD during night equaled 60 m) as the undisturbed situation. On average, we observed 13% of the population to dwell shallower compared to the undisturbed depth range during daytime, hence, facing lower than optimal salinity conditions (<13). A large proportion of 79% was observed deeper than the optimal depth range, hence, facing suboptimally low oxygen conditions. The individually experienced oxygen minimum of *P. acuspes* corresponded to 0.8 mL L^−1^.

## DISCUSSION

In this study, we combined observations of zooplankton individuals with a VPR and hydroacoustic estimates of predatory fish biomass and were able to unveil a small-scale DVM of Baltic *P. acuspes* in response to its planktivorous predators sprat and herring. DVM has been shown for many zooplankton of both, freshwater and marine species, in conventional studies before (e.g. Zaret and Suffern, 1976; Frost, 1988; Bollens *et al*., 1992) with vertical amplitudes ranging from a few to hundreds of meters (Hutchinson, 1967). However, we here were able to resolve diel vertical shifts of *P. acuspes* on very fine scales which might have been averaged out with traditional sampling approaches (Möller *et al*., 2012).

Furthermore, enabled by using an optical underwater observation technique, we additionally showed *in-situ* an immediate small-scale reaction of a zooplankton population to the behavior of the predator. Predation avoidance is the most likely reason for DVM of *P. acuspes* females in the Baltic Sea, resulting in the recorded bimodal pattern in the diel vertical distribution pattern in the deep basin.

Our observations strongly suggest that the copepods leave the food-rich marine snow layer in the halocline directly in response to the main predators sprat and herring (Möller *et al*., 2012). Furthermore, experimental studies have shown that Pacific herring exhibit a highly significant preference for ovigerous over non-ovigerous adult copepods (Bollens and Frost, 1991) and herring in the Baltic are likewise known to feed on *P. acuspes* with a preference for females over males (Flinkman *et al*., 1998; Bernreuther *et al*., 2013). Although we are confident in our results, the ultimate evidence that predation induces the DVM would be a control situation with no predators present during daytime and consequently no occurrence of DVM. We unfortunately did not encounter such a situation yet.

An experimental evidence confirming this assumption would require methods like *in-situ* mesocosm experiments, which have successfully been applied in freshwater habitats to study predator effects (e.g. Sell, 2000b) and DVM triggers (Fischer *et al*., 2006), but are very demanding to use in the marine environment and have to our knowledge only been used in coastal areas (e.g. Stibor *et al*., 2004; Sommer *et al*., 2004; Taucher *et al*., 2017), but not offshore, let alone over depth intervals of 60–80 m.

Additionally, the trigger for the migration still remains unclear and waits for experimental verification. A candidate trigger is mechanoreception that is widely used by copepods to detect and react to hydrodynamic disturbances created by approaching predators (Bollens *et al*., 1994; Buskey *et al*., 2011). Furthermore, the chemoreception of kairomones released by fish predators is another commonly discussed trigger (Cohen and Forward, 2009).

Baltic *P. acuspes* is a marine species with preferences for high salinities and low temperatures and, hence, we observed egg-carrying females in the deep and saline bottom water confirming earlier studies showing that salinity is the main factor driving the vertical distribution (Hansen *et al*., 2006; Renz and Hirche, 2006). Most previous studies have been using conventional net sampling and due to the coarser vertical resolution were not able to show DVM for this key species of the Baltic Sea food web. However, despite its ability to identify individual planktonic animals on small spatial and temporal scales, species identification still remains problematic based on VPR-derived images. We focused our study exclusively on ovigerous adult females of *P. acuspes* that carry their eggs until hatching in a conspicious external egg sac (Fig. 1). This special life-history feature allowed a discrimination from other species and developmental stages, usually impossible with VPR-derived pictures, since *P. acuspes* females are the only medium sized copepods in the central Baltic Sea carrying one egg sac (Möller *et al*., 2012). This procedure may introduce a bias in the perception of the DVM when interpreted as characteristic of the whole adult population since it ignores non-reproducing females and male *P. acuspes* (Bollens and Frost, 1989; Holliland *et al*., 2012). We, however, recorded the part of the population that is mainly important for producing the subsequent generation. Net sampling on the other side would have resulted in a loss of the egg sacs and, hence, a discrimination between egg-carrying and not reproducing females would have been impossible.

Our study provides *in-situ* evidence of strong individual variation in a marine copepod species´ DVM. Most of the *P. acuspes* females showed a general trend of a downward migration during day, but some individuals responded to the presence of predators with an upward movement towards the surface. Previous experimental studies have shown stage-specific differences in behavior due to size or reproductive status (Holliland *et al*., 2012). Bollens and Frost (1991) revealed that ovigerous copepod females of *Euchaeta elongata* differ in their vertical migration behavior compared to non-ovigerous adults by remaining at depth both day and night to avoid visually orienting predators. However, we here show that behavioral responses additionally can also vary between individuals of one developmental stage. The two different behavioral modes of ovigerous *P. acuspes* in the Baltic indicate that different predator avoidance strategies might exist in a population. The regular DVM of most of the individuals might represent an evolutionary developed behavior imprinted in the population based on genetic selection (Stich and Lampert, 1981). The upward movements of single individuals are possibly a more spontaneous responsive escape behavior. Recent research has shown that the decision to migrate might depend on individual traits, such as size, sex or conditional state (Olsson *et al.,* 2006; Brodersen *et al.,* 2008; Hansson and Hylander 2009). The direction of the response in our case is likely to depend on the position of the copepods in the water column relative to their predators or even direct encounters.

In our opinion, our VPR observations deliver strong evidence for individual variability in behavior of Baltic *P. acuspes* females. However, the ultimate evidence for individual-based behavior would be to track many animals over time which is impossible with small meso-zooplankton *in-situ*. Laboratory experiments would be necessary allowing individual tracking with camera systems, but the natural setting observed in our study will be difficult to reproduce. We were able to observe individual variability due to the ability of the VPR to conduct a continuous sampling over a long period of time and on small spatial scales at a stationary sampling site that serves as an comparable method for tracking individuals, although not following it. Furthermore, we observed the behavioral pattern repeatedly during two different cruises making an observation based just on chance unlikely.

Our results indicate that the risk of predation during daytime forces large parts of the *P. acuspes* adult female population out of their optimal habitat. The latter, which they inhabit during night, is characterized by suitable salinity and oxygen conditions (Renz and Hirche, 2006) as well as accumulations of marine snow where they find food (Bochdansky *et al*., 2010; Möller *et al*., 2012). Hence, during daytime most of the population is forced into low oxygen zones, which can function as a refuge against predation (Larsson and Lampert, 2011). Because *Pseudocalanus* is not able to support their metabolism under low-oxygen conditions through the synthesis of respiratory pigments like hemoglobin, which has only exceptionally been observed in copepod species (Sell, 2000a), this might have detrimental effects on the females and their offspring due to increased respiration rates (Marcus *et al*., 2004). Conversely, the part of the population that avoids predation by moving towards the surface encounters potentially too low salinity conditions which have negative consequences for their physiological performance (Gaudy *et al*., 2000; Renz and Hirche, 2006). The animals may, however, account for the period of sub-optimal conditions during day by making use of their saline, oxygenated, and food-rich habitat undisturbed by predators during night. However, these short-term detrimental effects might hinder a long-term optimization through adaptation which might be critical with respect to the current climate-induced reduction of major inflow events of saline and oxygenated water from the North Sea and a further narrowing of the vertical habitat of *P. acuspes* (Möller *et al*., 2015).

## CONCLUSION

In summary, combining observations of zooplankton individuals with the VPR and hydroacoustic estimates of predatory fish biomass, we here show (i) a small-scale DVM of ovigerous Baltic *P. acuspes* in response to its main predators sprat and herring, (ii) *in-situ* observations of a direct short-term reaction of the prey to the arrival of the predator and (iii) *in-situ* evidence of strong individual variation in this adaptive behavior. Furthermore, we show that this adaptive behavior to predation has the cost of accepting unfavorable conditions in terms of physical habitat parameters and feeding conditions. Thus, the increase in planktivorous predator populations through trophic cascading (Casini *et al*., 2008; Möllmann *et al*., 2008) may have caused direct consumptive effects on the population of *P. acuspes*, but as shown here indirect non-consumptive effects as well.
